# 腺嘌呤碱基编辑器纠正人胚胎G6PC3基因致病突变

**DOI:** 10.3760/cma.j.issn.0253-2727.2023.04.008

**Published:** 2023-04

**Authors:** 嫚 洪, 萍 王, 陶 上官, 广磊 李, 瑞鹏 边, 畏 何, 雯 江, 洁平 陈

**Affiliations:** 1 贵州大学医学院，贵阳 550025 Guizhou University Medical College, Guiyang 550025, China; 2 陆军军医大学第一附属医院（西南医院）血液内科，重庆 400038 Department of Hematology, the First Affiliated Hospital of Army Medical University（Southwest Hospital）, Chongqing 400038, China; 3 陆军军医大学第一附属医院（西南医院）生殖医学科，重庆 400038 Department of Reproductive Medicine, the First Affiliated Hospital of Army Medical University（Southwest Hospital）, Chongqing 400038, China; 4 广州医科大学第三附属医院生殖医学系，广州 510150 Department of Reproductive Medicine, Third Affiliated Hospital of Guangzhou Medical University, Guangzhou 510150, China

**Keywords:** 基因，G6PC3, 碱基编辑, 胚胎纠正, 重型先天性中性粒细胞减少症, Gene, G6PC3, Base editing, Embryo correction, Severe congenital neutropenia

## Abstract

**目的:**

研究腺嘌呤单碱基编辑器（ABE7.10）对人G6PC3基因致病性突变纠正的可行性。

**方法:**

构建不同sgRNA的表达载体，分别在人HEK293T突变模型和人类胚胎中尝试纠正的可行性和编辑效率，通过深度测序进行脱靶分析。

**结果:**

①成功构建了G6PC3^C295T^的突变细胞模型。②构建了三个不同的Re-sgRNA，并在G6PC3^C295T^突变细胞模型中实现了纠正，碱基纠正效率为8.79％～19.56％。③人致病胚胎实验证明ABE7.10能有效纠正突变碱基，但同时在其他位置也发生了碱基编辑事件。④深度测序分析显示，对32个潜在脱靶位点检测，除1个非编码区位点转换效率为1.03％外，其余均低于0.5％，具有较高的安全性。

**结论:**

该研究提出了一种新的基于人类致病胚胎的碱基纠正策略，但也产生了一定的非目标位点编辑，后续还需在PAM位点或者编辑器的窗口上进行进一步研究。

## Abstract (en)

**Objective:**

To determine whether the adenine base editor（ABE7.10）can be used to fix harmful mutations in the human G6PC3 gene.

**Methods:**

To investigate the safety of base-edited embryos, off-target analysis by deep sequencing was used to examine the feasibility and editing efficiency of various sgRNA expression vectors. The human HEK293T mutation models and human embryos were also used to test the feasibility and editing efficiency of correction.

**Results:**

①The G6PC3^C295T^ mutant cell model was successfully created. ②In the G6PC3^C295T^ mutant cell model, three distinct Re-sgRNAs were created and corrected, with base correction efficiency ranging from 8.79％ to 19.56％. ③ ABE7.10 could successfully fix mutant bases in the human pathogenic embryo test; however, base editing events had also happened in other locations. ④ With the exception of one noncoding site, which had a high safety rate, deep sequencing analysis revealed that the detection of 32 probable off-target sites was <0.5％.

**Conclusion:**

This study proposes a new base correction strategy based on human pathogenic embryos; however, it also produces a certain nontarget site editing, which needs to be further analyzed on the PAM site or editor window.

重型先天性中性粒细胞减少症（SCN）是一种以骨髓和外周血中成熟中性粒细胞缺乏为特征的异质性遗传性综合征[1]，患病率约为6/100万[2]。该疾病与众多基因突变有关，如ELANE、HAX1、WAS、G6PC3等[3]。其中，由G6PC3基因突变引起的称之为严重先天性中性粒细胞减少4型（SCN4）[4]。目前SCN4报道的突变形式主要有单个碱基突变和小片段缺失[5]，导致酶功能丧失。外显子6处单个碱基突变最多，外显子2鲜有报道[4]。该病常发生于婴幼儿，严重可导致死亡。单碱基编辑器是一类可对特定碱基进行转换的工具[6]–[7]。其中，ABE7.10经过了七轮改进，相比于第一代ABE1.2效率提高了7.4倍[7]；在HEK293T细胞中转染48 h的靶突变效率在10％～35％[7]，其窗口在4～7位[8]，便于进行单碱基编辑。现今，已报道的点突变产生的疾病约占人类遗传疾病总数的2/3[9]，修复单碱基突变有望成为治愈该类疾病最为有效的突破口。目前，对于SCN4致病胚胎的纠正编辑还鲜见报道。使用单碱基编辑器校正突变位点可能是G6PC3介导SCN4的潜在治疗方法。本研究中我们利用含有G6PC3基因单点突变（c.295C>T，碱基突变将产生无义突变）的精子细胞，通过体外受精的方式获得携带杂合突变的胚胎模型，探索使用ABE7.10系统在人类胚胎（<14 d）中纠正突变的效率，探索基因编辑治疗致病胚胎的方法。

## 材料与方法

一、主要材料和试剂

Lipofectamine™ 2000瞬时转染试剂、DMEM、PBS、Opti-MEM™、胰蛋白酶、青霉素-链霉素、短片段RNA体外转录试剂盒、mMESSAGEmMACHINE^®^ T7 Ultra Kit购于赛默飞世尔科技（中国）有限公司；嘌呤霉素、氨苄青霉素钠、氯化钠、胰蛋白胨、酵母粉、琼脂粉购于北京索莱宝科技有限公司；T4 DNA连接酶、Bsa Ⅰ酶、Bbs Ⅰ酶购于NEB（北京）有限公司；Phanta Max Super-Fidelity DNA Polymerase试剂盒、Discover-sc Single Cell WGA Kit、PCR清除试剂盒、FastPure Gel DNA Extraction Mini Kit、FastPure Plasmid Mini Kit购于南京诺唯赞生物科技股份有限公司；胎牛血清购于上海双洳生物科技有限公司；QIAprep Spin Miniprep Kit、血液/细胞/组织基因组DNA提取试剂盒购于北京天根生化科技有限公司；HEK293T细胞、U6质粒、pUC57质粒、SpCas9质粒由陆军军医大学第一附属医院血液病中心实验室保存；ABE7.10由上海科技大学黄行许实验室馈赠；实验所需引物和桑格尔测序均在重庆擎科生物科技有限公司进行；深度测序在重庆生命知源科技有限公司进行。

二、伦理申明

本研究获得了陆军军医大学第一附属医院伦理委员会的批准（批件号KY201921）。患者父亲在采集样本前签署了知情同意书。所有相关的临床和实验程序均在陆军军医大学第一附属医院进行。

三、质粒构建和体外转录

1. 质粒构建：合成用于突变和纠正的sgRNA寡核苷酸链（Oligo），将其克隆到pgl3-U6-sgRNA-Bsa Ⅰ ACCG puro表达载体上。简言之，用Bsa Ⅰ酶对U6质粒进行酶切，使用PCR清除试剂盒进行纯化，取U6纯化回收产物和Oligo退火产物用T4连接酶进行连接转化，使用Phanta Max Super-Fidelity DNA Polymerase试剂盒对重组质粒进行鉴定，待扩增结束后，取PCR扩增产物使用琼脂糖凝胶电泳检测，根据电泳条带确定阳性菌落，并将阳性菌落送往公司测序鉴定。参考正常的G6PC3基因外显子2处的碱基序列和精细胞发生突变的位点，请生物公司合成体外修复模板ssODN，其序列如下：5′-GGTGGGTCCATGAGTCTGGTTACTACAGCCAGGCTCCAGCCCAGGTTCACtAGTTCCCCTCTTCTTGTGAGACTGGTCCAGGTGGGAAGCCTCAAACATT-3′。

2. 体外转录：用Bbs Ⅰ酶将ABE7.10载体切开，使用mMESSAGE mMACHINE T7 Ultra Kit进行转录，将所有用于体外转录的sgRNAs克隆到一个带有T7启动子的pUC57-sgRNA表达载体中。然后，使用短片段RNA体外转录试剂盒对sgRNAs进行体外扩增和转录，随后使用QIAprep Spin Miniprep Kit进行纯化ABE7.10 mRNA和gRNAs，并在RNase-freeH_2_O中重悬，用Nanodrop 2000测定转录后的RNA浓度，RNA样品在用于显微注射前储存在−80 °C。

四、细胞培养和转染

在DMEM培养基中添加10％胎牛血清和1％青霉素链霉素，将HEK293T细胞置于培养基中并在37 °C恒温、5％CO_2_的细胞培养箱中培养。转染试剂使用方法按照制造商的协议进行（美国Invitrogen公司产品）。在转染前1天，将HEK293T细胞接种于6或12孔板上。细胞密度为60％～70％即可进行转染，在构建细胞突变体时，用Lipo2000将SpCas9、sgRNA和ssODN按照2∶1∶1的比例转染HEK293T细胞，48 h后用2 µg/ml嘌呤霉素筛选1周；在纠正突变体细胞时，用Lipo2000将ABE7.10、Re-sgRNA按2∶1比例转染293^C295T^突变体，48 h后提取细胞DNA鉴定，使用Edit R[10]分析测序数据。

五、有限稀释法挑取单克隆并鉴定

嘌呤霉素筛选1周后，收集部分细胞并使用血液/细胞/组织基因组DNA提取试剂盒提取DNA，使用Phanta Max Super-Fidelity DNA Polymerase试剂盒及引物对目标片段进行扩增，将扩增产物送往公司进行桑格尔测序，根据测序结果将目标位点处产生双峰的细胞群通过有限稀释法挑取单个细胞进行培养。简言之，将细胞制成细胞悬液后，对细胞进行计数，按照每96孔板接种60个细胞的比例进行稀释，稀释完成后转移至96孔板中，每孔添加培养基至150 µl。次日，在显微镜下观察贴壁细胞，标记只有一个细胞的小孔，每隔3 d换液1次，待细胞团生长至75％的平板面积后，转移至24孔板中并使用含有嘌呤霉素的培养基继续培养，收集部分细胞进行鉴定。

六、体外受精及显微注射

将用于本研究的卵母细胞通过玻璃化解冻后，置于G-IVF plus（瑞典Vitrolife公司产品）中培养2 h，同时解冻精子，所有卵细胞使用卵胞质内单精子显微注射技术（ICSI）授精，授精后将卵母细胞转至加盖矿物油的G-1 plus（瑞典Vitrolife公司产品）培养皿中，放入6％CO_2_、5％O_2_和89％N_2_的培养箱中继续培养。培养16～18 h后在显微镜下观察原核形成情况，并选择出现双原核的样本进行二次胚胎注射：将sgRNA溶液稀释为50 ng/ml，ABE7.10生成的mRNA稀释到100 ng/ml，继续使用ICSI方法将ABE7.10编辑系统和sgRNA混合物注射至接近双原核的位置，按照IVF实验室常规方法在胚胎培养基中保存3 d，然后收集整个胚胎进行实验。使用Discover-sc单细胞试剂盒扩增收集到的胚胎，以获得足够的DNA用于基因型鉴定。

七、脱靶分析和深度测序

使用web工具http://crispr.mit.edu/中Cas-OFFinder[11]预测了用于纠正G6PC3基因4个sgRNA的8个潜在脱靶位点，根据以下标准进行了筛选：①碱基错配数≤3；②碱基序列中至少包含一个A碱基。目标位点和脱靶位点的高通量DNA测序所需的PCR引物设计在位点周围的±150 bp处。使用Phanta Max Super-Fidelity DNA Polymerase试剂盒扩增所有的脱靶位点，将胶回收纯化后的PCR产物汇集在一起，使用Illumina NovaSeq6000（2×150）平台进行深度测序。使用BWA和PRISM工具对数据进行处理。

## 结果

1. G6PC3^C295T^ 细胞模型的建立：我们利用CRISPR技术构建与胚胎突变形式相同的细胞模型，用以在细胞系水平验证我们的编辑策略。首先，我们确认了精细胞G6PC3^C295T^位点的突变形式，提示供者为单等位基因的杂合突变（图1A），因此，预测用该精细胞将获得两种胚胎基因型，一种是单等位基因的杂合突变型，一种是正常胚胎。我们使用CRISPR系统结合外源修复模板（寡核苷酸链ssODN）的形式在细胞系中构建G6PC3^C295T^突变模型（图1B）。

首先，我们利用SpCas9、sgRNA和含有所需突变等位基因序列的ssODN供体，创建了一个细胞模型（图1B）。我们设计了一个sgRNA（图1C），编辑后测序，发现经sgRNA1转染后发生了切割，目标位点出现了明显的双峰（图1D）。分选单克隆后（图1E），通过鉴定发现单克隆编辑效率为2/15，共有11种基因型，以产生片段缺失为主，共计获得了两株与可能产生的单等位基因杂合突变型胚胎基因型一致的单克隆细胞系（图1F），最终我们选择该单克隆作为细胞模型（G6PC3^C295T^），进行下一步研究。

**图1:**
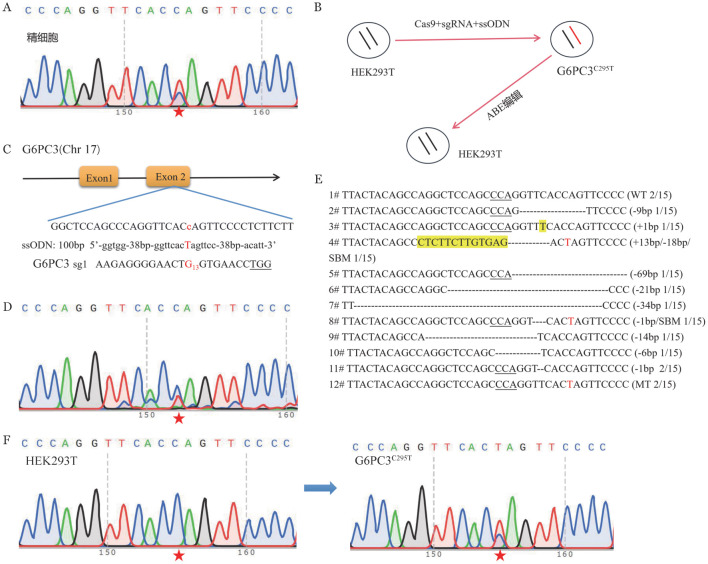
G6PC3^C295T^细胞模型的建立 A 携带者精细胞DNA测序色谱图（红色五角星所示为突变位点）；B 模拟和纠正细胞系中致病性突变的程序（第一步，使用CRISPR/Cas9结合ssODN使细胞产生同源重组修复，获得与胚胎一致的突变体；第二步，使用ABE单碱基编辑器结合具有修复的sgRNA对突变体进行校正）；C sgRNA及ssODN的碱基序列；D 转染后细胞团测序色谱图（目标位点用红色五角星表示）；E 具有基因组编辑功能的单细胞克隆的基因型分析（被修改后的碱基用红色字母表示，碱基插入用黄色表示，缺失用符号-表示，单个碱基突变用SBM表示）；F 野生型细胞与突变细胞测序色谱图（目标位点用红色五角星表示）

2. 突变体293T细胞系致病点突变的纠正：获得G6PC3^C295T^细胞模型（简称293^C295T^）后，我们在细胞模型上验证我们的编辑策略。由于其突变形式是C>T，我们需要用ABE7.10编辑工具对其互补链进行A>G的编辑，从而纠正细胞模型中的293^C295T^致病性突变。为此，我们设计了3个校正sgRNA（Re-sgRNA，以下简称Re-sg1、Re-sg2、Re-sg3）（图2A）。用Re-sgRNA和ABE7.10表达质粒同时转染293^C295T^细胞。结果显示，与突变型293^C295T^细胞比较，实验组中检测到目标位点碱基T的含量发生了不同程度的下降（图2B），293^C295T^细胞的T碱基占比为60.78％，实验组Re-sg1、Re-sg2、Re-sg3的T碱基占比分别为51.67％、48.89％、55.44％（图2C），表明发生了纠正。之后，我们根据T碱基所占百分比，根据1-实验组T碱基占比/对照组T碱基占比计算出3个Re-sgRNA（A13、A8、A1）在致病位点的编辑效率分别为14.99％、19.56％和8.79％。在Re-sgRNA3细胞团的测序图谱中显示，在目标位点附近出现了其他编辑位点（图2D），Re-sgRNA1和Re-sgRNA2并未出现该种现象。为检验ABE7.10编辑后产生副产物，我们将PCR产物经TA克隆并测序，确认产生突变的结果。我们分别对实验组和对照组的PCR产物经TA克隆后各挑取30个菌落进行测序。结果表明，实验组中目标位点T碱基所占的比例相较于对照组出现了明显的下降（图2E），对照组中突变型占比56.67％（17/30），Re-sgRNA1和Re-sgRNA3突变型占比分别为27.59％（8/29）、25.00％（7/28）。在这些编辑过的克隆中，仅有1个在除目标位点处的其他位置有T-to-C转换（图2F）。结果表明，ABE7.10编辑工具能对该致病性突变进行纠正。

**图2:**
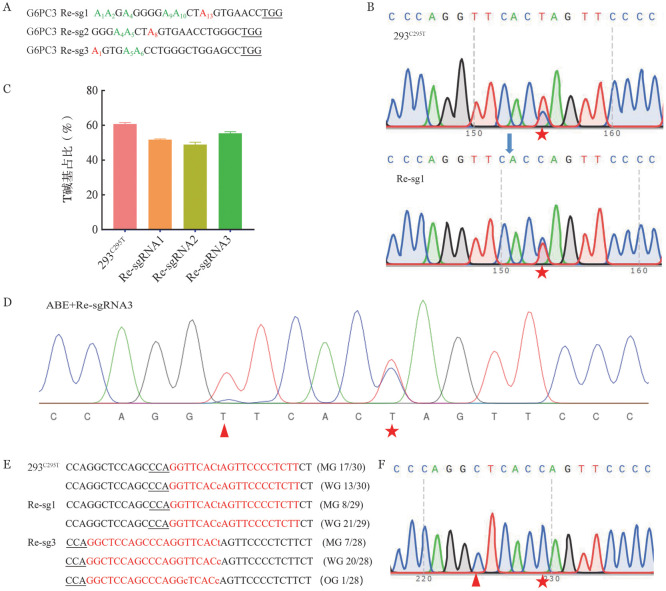
在G6PC3^C295T^细胞中通过碱基编辑纠正致病性G6PC3^C295T^突变 A 纠正sgRNA的碱基序列；B PCR产物测序的代表性色谱图（目标位点用红色五角星表示）；C 目标位点处T碱基的占比（实验重复3次）；D Edit R分析后测序色谱图（目标位点用红色五角星表示，非目标位点用红色三角形表示）；E TA克隆后单菌落测序分析（对B图的PCR产物的TA克隆进行DNA测序分析，MG：突变基因型；WG：野生基因型；OG：其他基因型）；F Re-sg3的其他基因型测序色谱图（目标位点用红色五角星表示，非目标位点用红色三角形表示）

3. 对人类胚胎中G6PC3^C295T^突变的纠正：我们测试了在人类胚胎中纠正G6PC3^C295T^突变的情况。将成熟卵母细胞用携带G6PC3^C295T^突变的精子进行ICSI授精（图3A），获得携带杂合突变的胚胎。16～18 h后，观察胚胎的情况，选择二原核受精卵，使用ICSI将100 ng/ml ABE7.10编辑系统和50 ng/ml sgRNA混合物注射进二原核受精卵。作为对照，将携带者精细胞与正常卵细胞进行受精并培养。我们总共获得了3个对照胚胎和18个实验胚胎。2 d后，扩增收集的胚胎，测序后如图3B所示，实验组G6PC3基因目标位点碱基显示为C，而对照组3个胚胎中有1个目标位点T碱基含量接近50％，符合实验预期，表明ABE系统在人类二核胚胎目标位点中表现出有效编辑。

经Edit R分析后可知（图3C），受精后3 d内多数胚胎是属于八细胞胚胎阶段，根据胚胎中不超过十个细胞，突变基因型最小频率不超过5％，则认为完全纠正[12]。在18个测试组中，共有16个胚胎目标位点野生型达到了95％以上，除Re-sg2-1和Re-sg3-3分别为92％和82％（未达到95％），其纠正效率高达88.89％（16/18）。除了理想的纠正外，我们在非目标位点上检测到了明显的编辑现象（图3D），即出现了副产物，有44.44％的胚胎由无义突变转变成了错义突变，所编码的氨基酸从苯丙氨酸转换为脯氨酸或者亮氨酸（图3E）。

**图3:**
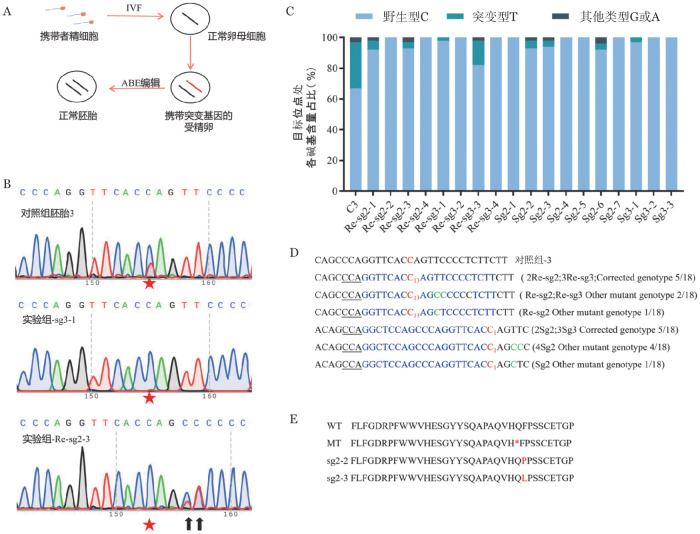
对人类胚胎中G6PC3致病性突变的修正 A 用ABE7.10纠正人类胚胎中致病性突变的实验程序示意图；B 对照组和纠正后的人类胚胎PCR产物测序的代表性色谱图（目标位点用红色五角星表示；其他突变位点用黑色箭头表示）；C 使用Edit R进行的基因分型分析图（对照组胚胎3用C3表示）；D 胚胎纠正及产生副产物情况；E G6PC3部分碱基的氨基酸翻译（终止密码子用*表示，脯氨酸用P表示，亮氨酸用L表示）

4. 深度测序进行脱靶分析：为进一步验证纠正胚胎的准确性，我们使用CasOFFinder预测工具来筛选可能的脱靶位点，即与sgRNA有同源性的基因组位点。基于筛选标准，我们对sgRNA2、sgRNA3、Re-sgRNA2、Re-sgRNA3分别进行了脱靶验证，每个sgRNA选取8个不同的脱靶序列，共计32个序列，并根据对应的胚胎进行潜在脱靶序列的扩增。我们分别扩增了实验组和对照组的32个潜在脱靶序列。每个序列的PCR产物均匀混合后进行深度测序。我们检测sgRNA 1-10位置（从远离PAM端开始计算）内的腺嘌呤位点的脱靶频率（如图4A和4B），共计61个位点。深度测序显示，在61个检测位点中，有47个样本与对照组相比，在编辑窗口内或附近的A/G转换无任何迹象。除OTS11G5以外，其余的转换效率均低于0.5％（OTS11G5位点A>G的转换效率为1.03％）。该位点在LOC124906221上，是智人物种中的非编码RNA。OTS11位于LOC124906221基因上游1.2 kb的基因间区域，根据Gene Cards数据库，暂未发现其关于正常人体组织中mRNA表达的数据以及蛋白质数据。

**图4:**
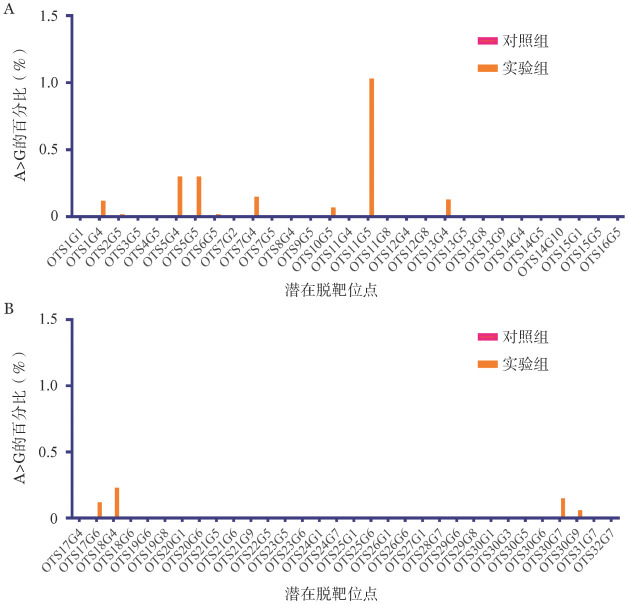
纠正和对照的人杂合子胚胎的脱靶分析 A OTS1G1-OTS16G5位点的深度测序结果；B OTS17G4-OTS32G7位点的深度测序结果

## 讨论

在本项研究中，我们成功地从人类细胞中建立突变细胞系，并在细胞和胚胎层面修复了致病突变。对于单个碱基编辑，ABE7.10产生副产物的情况更少，精准度较高，细胞中最高为19.56％，胚胎中则达到了88.89％。其次，细胞和胚胎中编辑效率也存在明显的差异。我们在细胞中使用质粒转染，胚胎中使用显微注射RNA，这提示我们不同的进入方式或者不同的sgRNA可能会对编辑效率造成一定的影响。此外，有研究表明，用ABE系统编辑基因组后，没有发现明显的脱靶[13]。我们对编辑后的胚胎进行脱靶检测，证实了ABE7.10应用在胚胎中的低脱靶率，提示其安全性。

一般来说，在绝大多数哺乳动物细胞中，以非同源末端连接（NHEJ）为主，而同源重组（HR）则通常只在分裂的细胞中发挥修复作用，且依赖于多种主要在细胞S期和G_2_期表达的蛋白[14]。NHEJ修复方式随机，它是强行的将DNA两端连接起来，由此很容易产生indels，造成突变结果的不确定和不可预测性。在细胞中，我们使用CRISPR/Cas9系统进行单碱基的编辑是低效的，细胞单克隆鉴定的数据显示，我们通过结合外源ssODN进行HR修复，虽可以精确地将所需突变引入，但用HR的方式获得突变株的效率非常低，与此同时仍产生了很多缺失、插入等突变，再次证明CRISPR编辑结果的不可控性。

我们的结果显示，在胚胎中可以纠正目标突变，但我们也应看到，ABE7.10的编辑窗口为4～7位，在编辑后会产生一定的副产物。因此，更加精准的编辑方法，或编辑窗口更小的编辑工具，可能会减少这方面的不利因素。已有研究显示，使用不同的碱基编辑器变体对超过3 000个与疾病相关的snv进行了纠正，准确率超过90％[15]。也可以从sgRNA出发，设计Imperfect guide-RNA（igRNA），该方法可大大增加单碱基编辑比例，提高了编辑效率[16]。

与以往的策略相比，对胚胎进行单碱基编辑策略具有几个重要的优势：①ABE编辑器不会造成双链断裂，脱靶效率低，安全性较高；②直接对胚胎进行编辑，可从根本上阻断基因传递下去，也会减少疾病给个体带来的痛苦；③直接对突变碱基加以纠正，恢复蛋白的功能，真正从根源上治疗该疾病。据我们所知，我们的研究是ABE7.10介导SCN4治疗的第一个例子，该策略不仅为治疗SCN4疾病，还为其他遗传病的治疗提供了一种新的思路。

因此，本研究提出了一种新的基于人类致病胚胎的碱基纠正策略。它具有有效性、精确性和安全性。值得注意的是，我们的编辑工具受限于PAM位点，设计的两个sgRNA包含的目标位点均未在编辑窗口中，而且，产生了一定的副产物，造成了编码氨基酸的改变，可能同样会影响该基因的功能。理论上这些限制可以通过使用基于新的Cas变体的碱基编辑器或者sgRNA的优化来规避，使绝大多数致病性转化突变具有靶向性[14]。后续可尝试采用现有开发的编辑器变体[17]–[18]或者开发出不受PAM位点限制且精确编辑的碱基编辑器。单碱基编辑器距离临床应用还需要更多的研究，以满足最高的安全性和有效性标准。
